# The Inhibitory Effects of NCT503 and Exogenous Serine on High-Selenium Induced Insulin Resistance in Mice

**DOI:** 10.3390/nu17020311

**Published:** 2025-01-16

**Authors:** Shuo Zhan, Jianrong Wang, Mingyu Zhu, Yiqun Liu, Feng Han, Licui Sun, Qin Wang, Zhenwu Huang

**Affiliations:** 1 National Institute for Nutrition and Health, Chinese Center for Disease Control and Prevention, Beijing 100050, China; zs_peipei@outlook.com (S.Z.); jianrongwang666@163.com (J.W.); z18846757274@163.com (M.Z.); liuyq@ninh.chinacdc.cn (Y.L.); hanfeng@ninh.chinacdc.cn (F.H.); sunlc@ninh.chinacdc.cn (L.S.); 2 Key Laboratory of Public Nutrition and Health, National Health Commission of the People’s Republic of China, Beijing 100050, China

**Keywords:** high-Se, IR, SSP, PHGDH inhibitor, serine

## Abstract

Objective: This study aims to identify whether the development of insulin resistance (IR) induced by high selenium (Se) is related to serine deficiency via the inhibition of the de novo serine synthesis pathway (SSP) by the administrations of 3-phosphoglycerate dehydrogenase (PHGDH) inhibitor (NCT503) or exogenous serine in mice. Method: forty-eight male C57BL/6J mice were randomly divided into four groups: adequate-Se (0.1 mgSe/kg), high-Se (0.8 mgSe/kg), high-Se +serine (240 mg/kg/day), and high-Se +NCT503 (30 mg/kg, twice a week) for 5 months. The glucose tolerance test (GTT) and insulin tolerance test (ITT) were used to confirm the development of IR in mice with high-Se intake, and fasting blood glucose levels were measured monthly. The Se contents in plasma and tissues were detected by ICP-MS. The levels of insulin (INS), homocysteine (HCY), and serine in plasma were tested by ELISA. Western blot analyses were conducted to evaluate the protein expressions of glutathione peroxidase 1 (GPX1), selenoprotein P (SELENOP) and PHGDH, the PI3K-AKT-mTOR pathway, folate cycle (SHMT1, MTHFR), and methionine cycle (MS). Results: An IR model was developed in mice from the high-Se group with elevated fasting blood glucose and INS levels, impaired glucose tolerance, and reduced insulin sensitivity, but not in both the high-Se +serine group and the high-Se +NCT503 group. Compared with the high-Se and high-Se +serine groups, the expressions of GPX1 and SELENOP significantly decreased for the high-Se +NCT503 group in the liver, muscle, and pancreas tissues. The expression of PHGDH of high-Se group was significantly higher than that of the adequate-Se group in the liver (*p* < 0.05) and pancreas (*p* < 0.001). Also, the expected high expression of PHGDH was effectively inhibited in mice from the high-Se +serine group but not from the high-Se +NCT503 group. The expression of p-AKT (Ser-473) for the high-Se group was significantly lower than that of the adequate-Se group in the liver, muscle, and pancreas. Conclusions: The IR induced by high-Se intake in the body has been confirmed to be partially due to serine deficiency, which led to the initiation of SSP to produce endogenous serine. The supplementations of exogenous serine or inhibitors of PHGDH in this metabolic pathway could be used for the intervention.

## 1. Introduction

Selenium is an essential trace mineral with critical roles in maintaining physiological homeostasis. As a vital component of selenoproteins, such as glutathione peroxidases (GPX) and thioredoxin reductases, selenium protects cells against oxidative stress and contributes to redox balance [1]. It also plays a key role in thyroid hormone metabolism, where Se-dependent enzymes regulate the activation and breakdown of thyroid hormones, and in modulating immune responses and inflammatory processes.

Dietary selenium is derived from various natural sources, including Brazil nuts, seafood, organ meats, poultry, eggs, and cereals. However, the selenium content of foods varies significantly by geographic region due to differences in soil selenium levels. The recommended dietary allowance (RDA) for selenium is 55 µg/day for adults, with increased requirements for pregnant (60 µg/day) and lactating women (70 µg/day). The tolerable upper intake level (UL) is 400 µg/day, beyond which adverse effects may occur.

While selenium deficiency is rare in most healthy populations, certain groups—such as individuals consuming Se-poor diets, patients with gastrointestinal disorders, or those dependent on parenteral nutrition—are at increased risk. Selenium deficiency can lead to diseases, such as Keshan disease (cardiomyopathy) and Kashin-Beck disease (osteoarthropathy), as well as impaired immune function and thyroid dysfunction.

Conversely, excessive selenium intake poses significant health risks. Chronic or acute selenium toxicity (selenosis) can result from consumption exceeding the UL, particularly through over-supplementation or dietary excess. Symptoms of selenosis include garlic breath odor, brittle hair and nails, gastrointestinal distress, fatigue, irritability, and neurological damage. In severe cases, selenium toxicity can lead to organ failure or death.

Beyond its toxic effects, supranutritional selenium intake has been increasingly linked to metabolic disorders, including insulin resistance (IR) and type 2 diabetes (T2D). IR is an important factor in the progression of T2D [2]. Large-scale studies in North America have associated excessive selenium intake with increased diabetes risk, potentially due to oxidative stress and disruption of insulin signaling pathways. These findings highlight selenium’s dual role in health, where adequate levels are essential for metabolic and cellular functions, but excessive levels drive metabolic dysregulation.

As early as 2004, hallmark features of T2D, including pancreatic hypertrophy, hyperinsulinemia, hyperglycemia, and obesity were developed in GPX1-overexpressing transgenic mice fed with 0.4 mgSe/kg diet [3]. Interestingly, when these mice were switched to a low-Se diet (<0.02 mgSe/kg) [4], most symptoms, except for hyperinsulinemia, were partially alleviated or disappeared.

In 2010, another research team from Japan found that selenoprotein P (SELENOP), synthesized by the liver and secreted into the bloodstream, also interfered with the normal signaling of insulin and disrupted glucose metabolism in cultured hepatic cells and skeletal muscle cells [5]. In vivo, the same research team first injected purified human SELENOP directly into mice and observed the occurrence of IR, which was relieved by administering a neutralizing antibody against human SELENOP [6].

Meanwhile, IR models have been successfully developed in different animals by feeding with high-Se diets naturally [7]. For example, Vyacheslav et al. reported that feeding male C57BL/6J mice a 0.4 mg/kg Se diet led to hyperinsulinemia and reduced insulin sensitivity, accompanied by a slight increase in the expression of certain selenoproteins [8]. Similarly, Zeng et al. observed IR-like features in pregnant Wistar rats and their offspring when fed a 3 mg/kg Se diet, with a concurrent increase in GPX1 expression [9].

The mechanistic basis for Se-induced abnormal glucose metabolism was first proposed by Lei Xingen’s research group [10,11], who identified IR as a key feature. This was attributed to the disruption of insulin signaling pathways due to low hydrogen peroxide (H_2_O_2_) levels, a consequence of the overexpression of antioxidant selenoproteins, such as GPX1 [12].

We previously reported that serine enhances selenoprotein expression in cultured cells [13], and discovered that disruptions in endogenous serine synthesis significantly impact selenoprotein expression in maternal tissues and subsequent offspring development [14]. These findings were further validated in vivo using a high-Se-induced IR mouse model [15], suggesting a potential link to the SESAME complex, which comprises S-adenosylmethionine (SAM) and various enzymes involved in glycolysis, serine synthesis, folate cycle, and methionine cycle [16].

Excessive selenium in the body leads to the accumulation of hydrogen selenide (H_2_Se), which must be methylated by SAM to be efficiently excreted as CH_3_^−^SeSUG or (CH_3_)_3_Se^+^. Without adequate SAM, severe toxic effects can occur. It has been observed that selenium toxicity in animals depletes SAM, which in turn leads to the depletion of serine, as recent studies suggest that synthesizing a single SAM molecule may consume a great amount of serine [17,18].

This study aimed to observe the inhibition effect of exogenous serine or a PHGDH inhibitor (NCT-503) on the activation of the serine synthesis pathway (SSP) in mice fed with high-Se, which provided a possible intervention for T2D induced by high-Se.

## 2. Materials and Methods

### 2.1. Reagents

Glucose, insulin, serine, primary antibodies (β-actin, PI3K, GPX1, SELENOP, SELENON, SHMT1, and MS), and HRP-conjugated IgG were sourced from Sigma-Aldrich (Shanghai, China). P-Akt (Ser473, Thr308), PHGDH, and MTHFR antibodies came from Abcam (Shanghai, China); mTOR and Akt antibodies were from Cell Signaling Technology (Denver, CO, USA). Additional chemicals were obtained from Wako Pure Chemical and Hitachi Co. (Osaka, Japan). The PHGDH inhibitor NCT-503 was provided by Shanghai Haoyuan (Shanghai, China) (MedChem Express). The ECL solution, Blue Plus II Protein Marker, and EasySee Western Marker were acquired from TransGen Biotech (Beijing, China).

### 2.2. Experimental Groups and Diet Regimes

**Figure 1:**
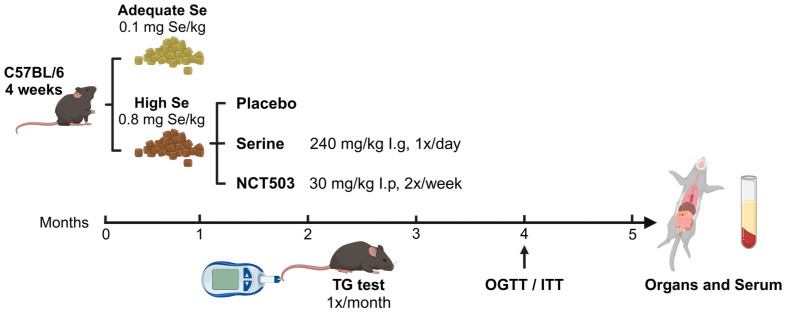
Animal experiment flowchart.

Figure 1 presents an outline of the animal experimental design. Forty-eight male C57BL/6J mice, aged 4 weeks and maintained at SPF status, were obtained from Beijing SBF Biotech Co., Ltd., Beijing, China, and housed under standard conditions. The temperature was maintained at 24 °C, and the relative humidity was kept at 55%. Following a one-week acclimation, mice were randomly assigned to four groups (n = 12): adequate-Se (0.1 mg Se/kg), high-Se (0.8 mg Se/kg), high-Se +serine (240 mg/kg/day), and high-Se +NCT503 (30 mg/kg, twice a week). The serine intervention group was fed with continuous 0.8 mg/kg Se and given serine by intragastric administration (240 mg/kg/day) [14,19]. NCT-503 was prepared by sequentially adding 10% DMSO (Sigma), 40% PEG300 (Sigma), 5% Tween-80 (Sigma), and 45% saline and administered intraperitoneally at a dose of 30 mg/kg twice weekly [20], with the injection volume kept below 150 μL per mouse.

The animal fodder was sourced under license SCXK (Beijing) 2024-0001. The composition of the food is provided in Table 1. The actual selenium contents measured by ICP-MS were 0.113 mg/kg for the adequate-Se group and 0.834 mg/kg for the high-Se groups. According to reference [7], glucose tolerance tests (GTTs) and insulin tolerance tests (ITTs) were conducted on mice starting from the fourth month. One month after IR was observed in the high-Se group, blood was collected from the retro-orbital sinus, mice were sacrificed, and target tissues were collected within 5 min to preserve their metabolic state. Tissue samples were stored at −80 °C.

### 2.3. Weekly Body Weight Monitoring in C57BL/6J Mice

Weekly body weight measurements were taken for C57BL/6J mice throughout the 5-month study period. Mice were weighed at the same time each week using a digital scale. The mice were fed diets containing 0.1 or 0.8 mg Se/kg, and body weight changes were recorded to assess the effects of different treatments.

### 2.4. Glucose Tolerance Test (GTT)

Measure blood glucose levels in the tail vein of mice using a blood glucose meter (Johnson & Johnson, Shanghai, China). Mice underwent a 12 h fast before receiving a glucose injection (1 mg/g) for the GTT, with an injection volume not exceeding 200 μL. Blood glucose levels were recorded at 0, 20, 30, 60, and 120 min.

### 2.5. Insulin Tolerance Test (ITT)

In the ITTs, mice were fasted for 12 h. Insulin (0.75 mU/g body weight) [21] was then administered intraperitoneally, with an injection volume not exceeding 200 μL. Tail vein blood glucose levels were measured at 0, 30, 60, 120, and 240 min.

### 2.6. Fasting Blood Glucose Test

Fasting blood glucose in the mice was monitored monthly. Following a 12 h overnight fast, tail vein blood samples were directly measured using a glucometer (Johnson & Johnson, Shanghai, China). Data were recorded as blood glucose concentrations at the time of measurement.

### 2.7. Selenium Detection of Plasma and Tissues in Mice

Plasma and tissue selenium levels were determined using microwave digestion with nitric acid, followed by analysis via inductively coupled plasma mass spectrometry (ICP-MS), as previously described [22].

### 2.8. Blood Biochemistry Analysis

Plasma levels of insulin (INS), homocysteine (HCY), and serine were measured using commercial ELISA kits, employing a double-antibody sandwich method for enzyme-linked immunosor. All assays were conducted following the manufacturer’s protocols.

### 2.9. PHGDH Enzyme Activity Assay

The enzyme activity of PHGDH was determined using an ELISA kit (Jiangsu Jingmei Biological technology Co., Ltd., Yancheng, China), following the manufacturer’s instructions. Samples were processed and analyzed as per the provided protocol. Absorbance (OD values) were measured at a wavelength of 450 nm using a microplate reader, and enzyme activity was calculated accordingly.

### 2.10. Western Blotting

Protein extraction, SDS-PAGE, membrane transfer, antibody incubation, and ECL detection were performed following standard protocols [23].

### 2.11. Statistical Analysis

Data are presented as mean ± Standard Deviation (SD). Graphs were generated using GraphPad Prism 9.0, and statistical analyses were performed with SPSS 20.0 software (SPSS Inc., Chicago, IL, USA). Group comparisons were evaluated using analysis of variance (ANOVA), followed by post hoc comparisons using the Sidak test. Prior to applying ANOVA, the assumptions of normality and homogeneity of variances were assessed using the Shapiro–Wilk test and Levene’s test, respectively. If the data violated the assumption of normality, the non-parametric Kruskal–Wallis test was employed. If heteroscedasticity was detected, Welch’s ANOVA was used instead of the standard ANOVA.

## 3. Results

### 3.1. Weekly Body Weight Monitoring

**Figure 2:**
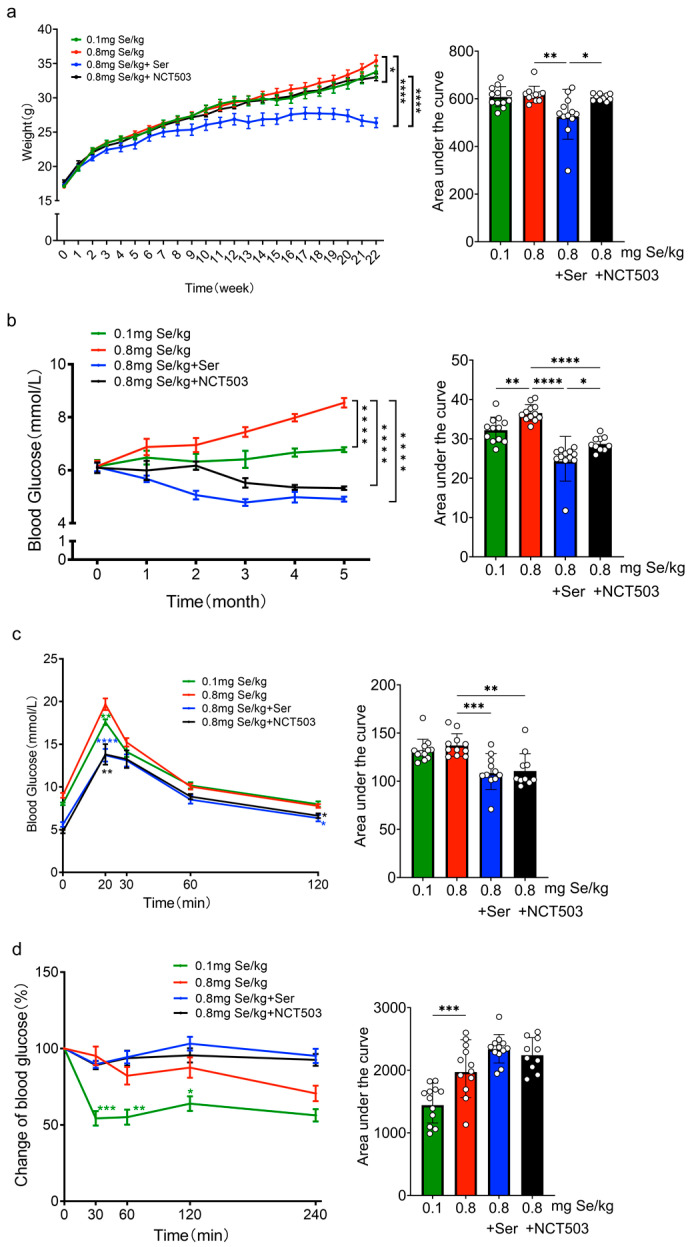
Effects of selenium levels (0.1, 0.8 mg Se/kg) and interventions (Ser, NCT503) on weight, glucose metabolism, GTT, and ITT in C57BL/6J mice. (**a**) Changes in body weight over 5 months. (**b**) Fasting blood glucose levels measured monthly. (**c**) Glucose tolerance assessed at the end of the fourth month. (**d**) Insulin tolerance evaluated at the end of the fourth month. (Data are expressed as mean ± SD, n = 10–12). (* *p* < 0.05, ** *p* < 0.01, *** *p* < 0.001, **** *p* < 0.0001; two-way ANOVA). Both Ser and NCT503 effectively mitigate Se-induced metabolic disturbances, with Ser showing a more substantial improvement in body weight and blood glucose regulation.

C57BL/6J mice were intervened, as shown in Figure 1 and Table S1, for 5 months, with body weight monitored weekly. Supplementation with Ser or NCT503 significantly reduced the Se-induced weight gain (**** *p* < 0.0001 and * *p* < 0.05, respectively), with the NCT503 group’s weight closer to that of the control group. The Ser group exhibited a markedly greater reduction in body weight than the NCT503 group (**** *p* < 0.0001) (Figure 2a).

### 3.2. Monthly Monitoring of Fasting Blood Glucose Levels

Mice were fed, as shown in Figure 2 and Table S1, for 5 months. Fasting blood glucose levels (tail vein blood after a 12 h fast) were monitored monthly. Mice of the 0.8 mg Se/kg group exhibited a gradual increase in blood glucose levels, reaching a peak at the fifth month (**** *p* < 0.0001) compared to the adequate-Se group. Supplementation with Ser or NCT503 improved fasting glucose levels gradually, which reached the lowest point at the fifth month compared to the high-Se group alone (**** *p* < 0.0001) (Figure 2b).

### 3.3. Glucose Tolerance Test

After a 4-month intervention, blood glucose levels were evaluated at specified time points following glucose injection (1 mg/g). Mice of the 0.8 mg Se/kg group exhibited significantly elevated blood glucose levels at 20 min compared to the 0.1 mg Se/kg group (** *p* < 0.01). Supplementation with Ser or NCT503 significantly improved glucose tolerance, resulting in lower blood glucose levels compared to the 0.8 mg Se/kg group alone (**** *p* < 0.0001 and ** *p* < 0.01, respectively) (Figure 2c and Table S1).

### 3.4. Insulin Tolerance Test

Mice were administered for 4 months. ITTs were performed, and the data were expressed as percentage changes from baseline. As illustrated in Figure 2d and Table S1, 30 min after insulin injection, the percentage change in blood glucose was significantly higher in the 0.8 mg Se/kg group compared to the 0.1 mg Se/kg group (*** *p* < 0.001). The elevated glucose response persisted at 60 and 120 min (** *p* < 0.01 and * *p* < 0.05, respectively), with no significant differences observed between the Ser and NCT503 groups.

### 3.5. High Se and Ser/NCT503 Supplementation Differentially Modulate Plasma Biochemical Markers

**Figure 3:**
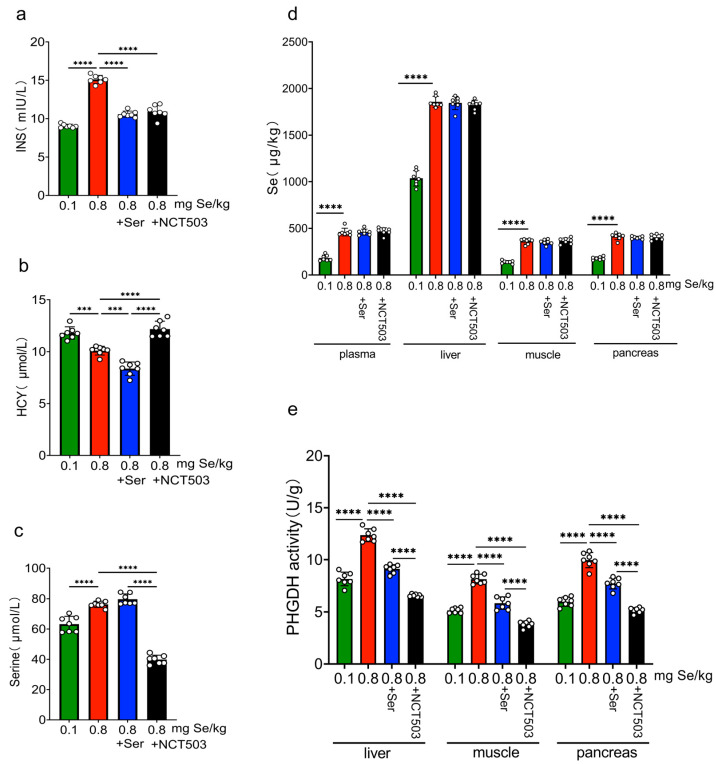
Effects of selenium levels (0.1 mg and 0.8 mg Se/kg) and interventions (Ser, NCT503) on plasma biochemical markers in C57BL/6J mice. (**a**–**c**) Changes in plasma biochemical markers: (**a**) insulin (INS, mIU/L), (**b**) homocysteine (Hcy, µmol/L), amd (**c**) serine (Ser, µmol/L). (**d**) Selenium levels in plasma, liver, muscle, and pancreas. (**e**) PHGDH enzyme activity in plasma, liver, muscle, and pancreas. (Data are expressed as mean ± SD, n = 7). (*** *p* < 0.001, **** *p* < 0.0001; two-way ANOVA). Supplementation with Ser and NCT503 modulates biochemical markers and PHGDH enzyme activity. Ser supplementation lowers INS and HCY levels, while NCT503 decreases INS and serine levels, and increases HCY levels. The NCT503 inhibitor has a stronger inhibitory effect on PHGDH enzyme activity.

As shown in Figure 3a and Table S2, INS levels were significantly elevated in the 0.8 mg Se/kg group compared to the 0.1 mg Se/kg group (**** *p* < 0.0001). Supplementary with Ser or NCT503 resulted in a significant reduction in INS levels (**** *p* < 0.0001). Figure 3b and Table S2 show that HCY levels were significantly lower in the 0.8 mg Se/kg group compared to the 0.1 mg Se/kg group (*** *p* < 0.001). Supplementation with serine further reduced HCY levels (**** *p* < 0.0001), whereas NCT503 intervention significantly increased HCY levels (**** *p* < 0.0001). In Figure 3c and Table S2, serine levels were significantly higher in the 0.8 mg Se/kg group compared to the 0.1 mg Se/kg group (*** *p* < 0.001). NCT503 supplementation significantly decreased serine levels (**** *p* < 0.0001) compared to the 0.8 mg Se/kg group, while serine supplementation slightly increased serine levels, although the change was not statistically significant.

### 3.6. Selenium Levels in Plasma, Liver, Muscle, and Pancreas

In the 0.8 mg Se/kg group, selenium concentrations in plasma, liver, muscle, and pancreas were markedly higher than those in the 0.1 mg Se/kg group (**** *p* < 0.0001). Selenium concentrations showed no significant variation with Ser or NCT503 supplementation (Figure 3d and Table S3).

### 3.7. PHGDH Activities in Liver, Muscle, and Pancreas

In the 0.8 mg Se/kg group, PHGDH enzyme activity in the liver, muscle, and pancreas was significantly higher than in the 0.1 mg Se/kg group (**** *p* < 0.0001). After supplementation with Ser or NCT503, PHGDH activity was markedly reduced (**** *p* < 0.0001). However, in the Ser intervention group, PHGDH activity was significantly higher than in the 0.1 mg Se/kg group (muscle: * *p* < 0.05; liver: ** *p* < 0.01; pancreas: ***** *p* < 0.0001). In contrast, PHGDH activity in the NCT503 intervention group was significantly lower than in the 0.1 mg Se/kg group (liver and muscle: **** *p* < 0.0001; pancreas: * *p* < 0.05). Furthermore, when comparing the Ser intervention group to the NCT503 group, PHGDH activity in the NCT503 group was also significantly reduced (**** *p* < 0.0001) (Figure 3e and Table S4).

### 3.8. Influence of Dietary Selenium Supplementation on the Expressions of Selenoproteins and Enzymes Related with Serine Metabolism in Mice Tissues

**Figure 4:**
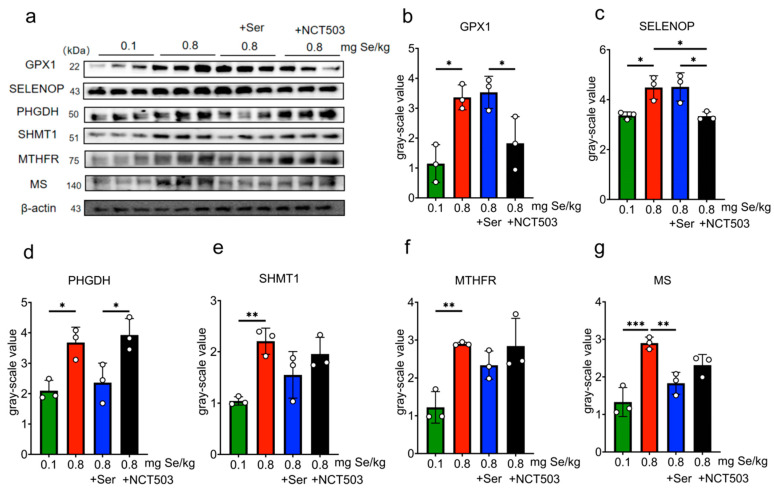
Effect of 0.1, 0.8 mg Se/kg and Ser or NCT503 intervention group on the Expressions of selenoproteins and enzymes in Liver Tissue. (**a**) WB images. (**b**–**g**) grayscale analysis. (Mean ± SD, n = 3, * *p* < 0.05, ** *p* < 0.01, *** *p* < 0.001; two-way ANOVA). NCT503 and Ser differentially modulate liver enzyme expression, with NCT503 enhancing PHGDH activity and reducing SELENOP, while Ser primarily decreases MS expression.

Liver. As shown in Figure 4 and Table S5, compared with the 0.1 mg Se/kg group, the expressions of GPX1, SELENOP, PHGDH, SHMT1, MTHFR, and MS were significantly higher for the 0.8 mg Se/kg group (MS, *** *p* < 0.001; SHMT1 and MTHFR, ** *p* < 0.01; GPX1, SELENOP and PHGDH, * *p* < 0.05). Mice in the Ser group showed a significant reduction in MS expression compared to the 0.8 mgSe group alone (MS, ** *p* < 0.01), while GPX1 and SELENOP expressions increased slightly, PHGDH, SHMT1, and MTHFR expressions decreased slightly, but these changes were not statistically significant. SELENOP expression (* *p* < 0.05) showed significant reductions in the NCT503 group compared to the 0.8 mgSe group. Compared to the Ser group, the expressions of GPX1 and SELENOP were lower (* *p* < 0.05), while the expressions of PHGDH were increased (* *p* < 0.05) in the NCT503 group.

**Figure 5:**
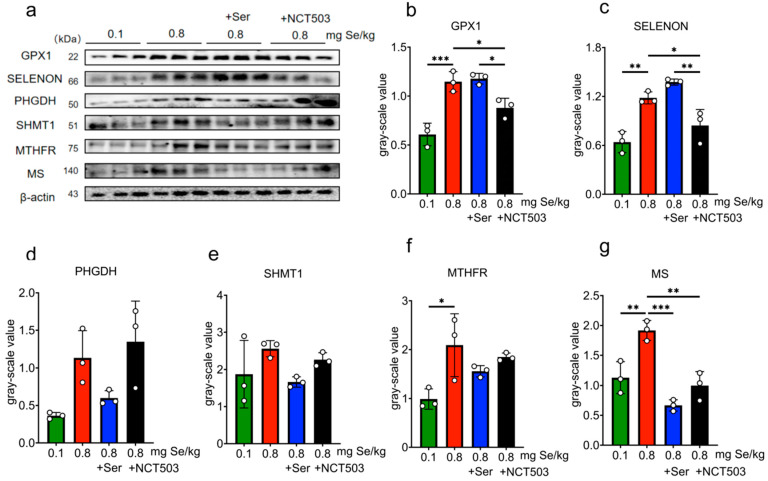
Effect of 0.1, 0.8 mg Se/kg and Ser or the NCT503 intervention group on the expressions of selenoproteins and enzymes in muscle Tissue. (**a**) WB images. (**b**–**g**) grayscale analysis. (Mean ± SD, n = 3, * *p* < 0.05, ** *p* < 0.01, *** *p* < 0.001; two-way ANOVA). NCT503 reduces GPX1 and SELENOP expression in muscle, while Ser mainly reduces MS expression.

**Muscle.** As shown in Figure 5 and Table S6, compared with the 0.8 mgSe group, the expressions of GPX1, SELENON, MTHFR, and MS were significantly higher for the 0.1 mgSe group (GPX1, *** *p* < 0.001; SELENON, MS, ** *p* < 0.01; MTHFR, * *p* < 0.05), while the expressions of PHGDH, SHMT1 increased slightly with no statistical significance. The expressions of MS in the Ser group showed significant reductions (MS, *** *p* < 0.001) compared with 0.8 mgSe alone. For the 0.8 mgSe group, the expressions of GPX1, SELENON, and MS decreased significantly compared to NCT503 group (MS, ** *p* < 0.01; GPX1 and SELENOP, * *p* < 0.05). Protein expressions were lower in the NCT503 group compared to the Ser group (SELENOP, ** *p* < 0.01; and GPX1, * *p* < 0.05).

**Figure 6:**
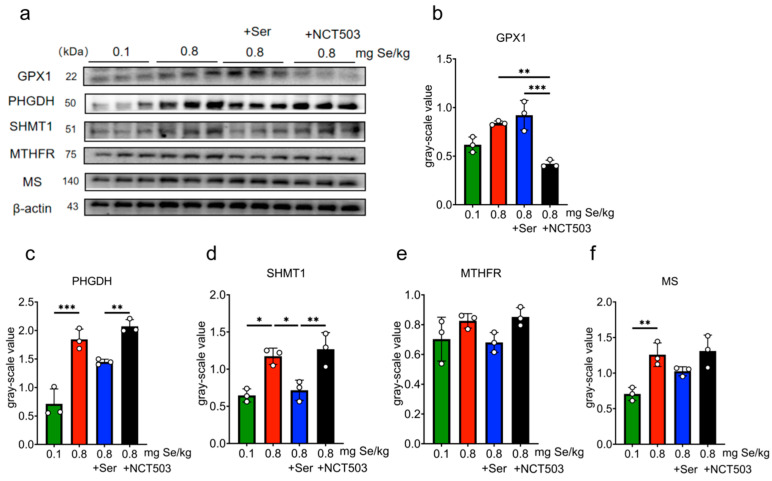
Effect of 0.1, 0.8 mg Se/kg and Ser or the NCT503 intervention group on the expression of selenoproteins and enzymes in Mouse pancreas Tissue. (**a**) WB images. (**b**–**f**) grayscale analysis. (Mean ± SD, n = 3, * *p* < 0.05, ** *p* < 0.01, *** *p* < 0.001; two-way ANOVA). NCT503 increases PHGDH expression in the pancreas, while Ser primarily reduces SHMT1 expression.

**Pancreas.** As shown in Figure 6 and Table S7, for the 0.8 mgSe group, the expressions of PHGDH, SHMT1, and MS were significantly elevated compared to the 0.1 mgSe group (PHGDH, *** *p* < 0.001; MS, ** *p* < 0.01; SHMT1, * *p* < 0.05). The expression of SHMT1 showed significant reductions for the Ser group compared to the 0.8 mgSe group alone (SHMT1, * *p* < 0.05), while the expressions of GPX1 increased slightly, and PHGDH, MTHFR, and MS decreased slightly with no statistical significance. Compared to the Ser group, the expressions of GPX1 (*** *p* < 0.001) decreased, and PHGDH, SHMT1 increased (PHGDH and SHMT1,** *p* < 0.01) for the NCT503 group.

### 3.9. Influence of Dietary Selenium Supplementation on the PI3K-AKT-mTOR Insulin Signaling Pathway in Mouse Tissues

**Figure 7:**
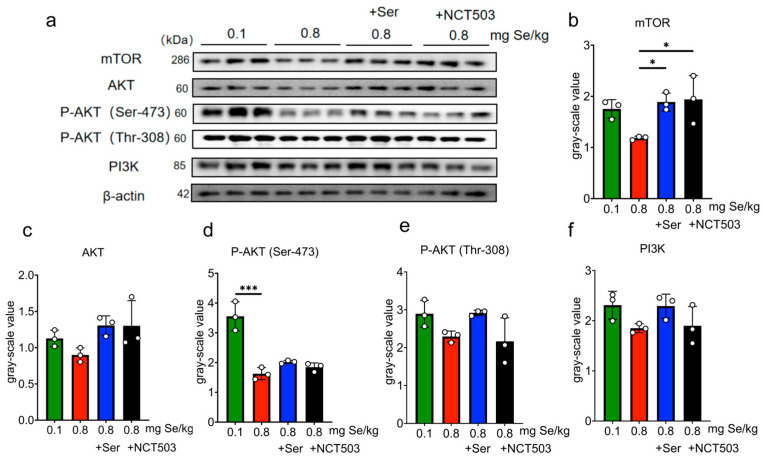
Effects of 0.1, 0.8 mg Se/kg, and Ser or the NCT503 intervention group on the PI3K-AKT-mTOR pathway in mice liver. (**a**) WB images. (**b**–**f**) Pathway grayscale analysis (Mean ± SD, n = 3, * *p* < 0.05, *** *p* < 0.001; two-way ANOVA). Ser and NCT503 supplementation modulate mTOR expression in the liver, with NCT503 and Ser both enhancing mTOR signaling.

Liver. As shown in Figure 7 and Table S5, the expression of phosphorylated Akt1 at Ser-473 (P-Akt (Ser-473)) was significantly lower in the 0.8 mg Se/kg group than in the 0.1 mg Se/kg group (*** *p* < 0.001); the expressions of mTOR, AKT, phosphorylated Akt1 at Thr-308 (P-Akt (Thr-308)), and PI3K were slightly decreased, but these changes were not statistically significant. Compared to the 0.8 mg Se/kg group, the expressions of mTOR increased significantly in the 0.8 mgSe/kg +Ser and 0.8 mg Se/kg +NCT503 groups (* *p* < 0.05). The expressions of AKT, P-Akt (Ser-473), P-Akt (Thr-308), and PI3K showed slight increases, but these were not statistically significant.

**Figure 8:**
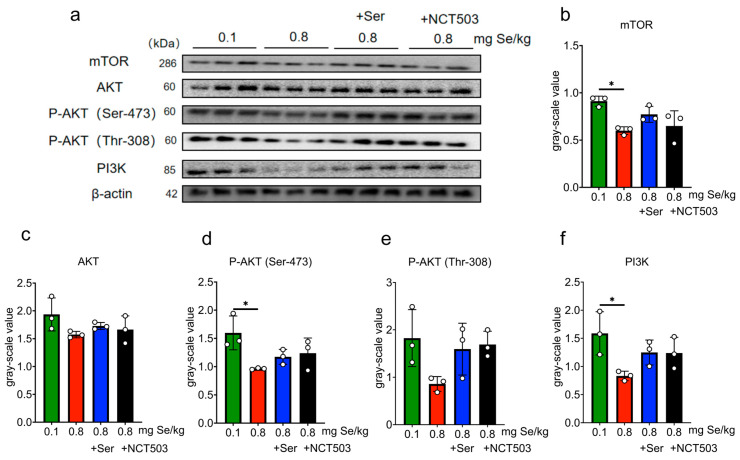
Effects of 0.1, 0.8 mg Se/kg, and Ser or the NCT503 intervention group on the PI3K-AKT-mTOR pathway in mice muscle. (**a**) WB images. (**b**–**f**) Pathway grayscale analysis (Mean ± SD, n = 3, * *p* < 0.05; two-way ANOVA). While Ser and NCT503 supplementation slightly increased Akt/mTOR pathway markers in muscle, these changes were not significant.

Muscle. As shown in Figure 8 and Table S6, mTOR, P-Akt (Ser-473), and PI3K expression levels were significantly lower in the 0.8 mg Se/kg group compared to the 0.1 mg Se/kg group (* *p* < 0.05). The expressions of AKT and P-Akt (Thr-308) were slightly decreased, but these changes were not statistically significant. Compared to the 0.8 mg Se/kg group, the expressions of mTOR, AKT, P-Akt (Ser-473), P-Akt (Thr-308), and PI3K in the 0.8 mg Se/kg +Ser or 0.8 mg Se/kg +NCT503 group showed slight increases, but these were not statistically significant.

**Figure 9:**
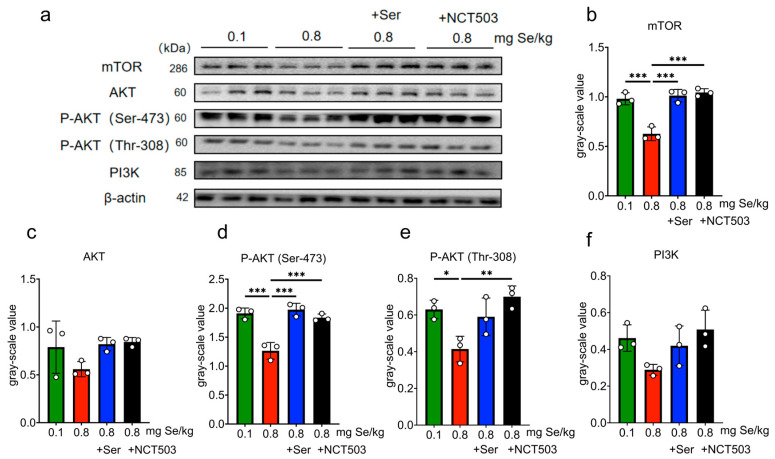
Effects of 0.1, 0.8 mg Se/kg, and Ser or the NCT503 intervention group on the PI3K-AKT-mTOR pathway in mice muscle pancreas. (**a**) WB images. (**b**–**f**) Pathway grayscale analysis (Mean ± SD, n = 3, * *p* < 0.05, ** *p* < 0.01, *** *p* < 0.001; two-way ANOVA). Both Ser and NCT503 supplementation restored Akt/mTOR signaling in the pancreas, with significant increases in mTOR and phosphorylated Akt levels compared to the high-Se group.

Pancreas. As shown in Figure 9 and Table S7, the 0.8 mg Se/kg group exhibited significantly reduced expressions of mTOR, P-Akt (Ser-473), and P-Akt (Thr-308) compared to the 0.1 mg Se/kg group (*** *p* < 0.001). The expressions of AKT and PI3K were slightly lower, but these changes were not statistically significant. The expressions of mTOR and P-Akt (Ser-473) increased significantly in the 0.8 mg Se/kg +Ser group (*** *p* < 0.001) compared to the 0.8 mg Se/kg group, while the expressions of AKT, P-Akt (Thr-308), and PI3K showed slight, non-significant increases. Similarly, compared to the 0.8 mg Se/kg group, the expressions of mTOR, P-Akt (Ser-473) (*** *p* < 0.001), and P-Akt (Thr-308) (** *p* < 0.01) were significantly increased in the 0.8 mg Se/kg +NCT503 group, while the expressions of AKT and PI3K showed slight, non-significant increases.

## 4. Discussion

At present, IR models have been developed through natural feeding with high-Se fodder in animals such as pigs, rats, mice, and chickens. Recently, we successfully re-established the high-Se-induced IR model in C57BL/6J mice fed with 0.4 mg Se/kg diet for 4 months based on the research [7]. In addition to the same occurrence of IR, we observed for the first time changes in the expressions of enzymes related to serine synthesis and metabolism [14].

Given the substantial evidence that excessive selenium intake beyond physiological needs raises the risk of diabetes in individuals, it mainly comes from two intervention cohort studies of selenium supplementation to prevent cancer in North America, and the total daily average selenium intake of subjects in the two cohort studies is 330 μg per day. In this study, the selenium content in the high selenium feed given to C57BL/6J mice increased from 0.4 mgSe/kg (equivalent to a daily selenium intake of 200 μg for adults) to 0.8 mgSe/kg (equivalent to a daily selenium intake of 400 μg for adults).

Excessive selenium intake (0.8 mg Se/kg) significantly disrupted glucose homeostasis, as evidenced by elevated fasting blood glucose levels, increased insulin secretion, impaired glucose tolerance, and reduced insulin sensitivity (Figure 2b–d and Figure 3a). Notably, mice in the high-Se group exhibited a significantly greater final body weight compared to the two intervention groups, while the high-Se +serine group showed the lowest body weight among all groups (Figure 2a). This unexpected observation suggests a complex interaction between selenium and serine in energy metabolism. The specific mechanisms underlying this weight reduction remain to be elucidated. Additionally, selenium concentrations in plasma and tissues, as well as plasma serine levels, were markedly elevated in the high-Se group relative to the adequate-Se group (Figure 3c,d), implying systemic selenium accumulation under high-Se conditions.

Consistent with prior studies [14], we observed a pronounced upregulation of GPX1, SELENOP, and SELENON across the liver, muscle, and pancreas in mice exposed to a high-Se diet (Figure 4, Figure 5 and Figure 6). These selenoproteins play pivotal roles in redox homeostasis, and their coordinated upregulation underscores the cellular need to counteract Se-induced oxidative damage. Importantly, serine supplementation enhanced the expression of GPX1 and SELENOP in the liver and SELENON in muscle, suggesting a synergistic interaction between serine and selenium in augmenting antioxidant defenses. This finding aligns with our previous research [12] and highlights serine’s potential as a metabolic modulator in the context of selenium exposure.

PHGDH, the first rate-limiting enzyme in the SSP, is high-expressed in various metabolic disorders, including cancer and glucose metabolism [24,25,26], to produce serine supplying one-carbon unit for the synthesis of nucleotide to meet the need of tumor cell growth. In this study, the protein expression and enzyme activity of PHGDH were significantly increased in the high-Se group and were inhibited by exogenous serine (Figure 3e, Figure 4d and Figure 6c). The content of serine decreased for the high-Se +NCT-503 group (Figure 3c). We observed that NCT-503 did not suppress PHGDH protein expression in the liver, muscle, or pancreas (Figure 4d, Figure 5d and Figure 6c), but significantly inhibited PHGDH enzyme activity (Figure 3e). NCT503 is designed as a specific inhibitor targeting the active site of PHGDH, by directly binding to the active center of PHGDH, without reducing its expression through changes in protein stability or degradation rate [27]. Studies reported that NCT-503 inhibited PHGDH enzyme activity without affecting PHGDH protein expression [20,28,29], which aligns with the experimental results we observed.

**Figure 10:**
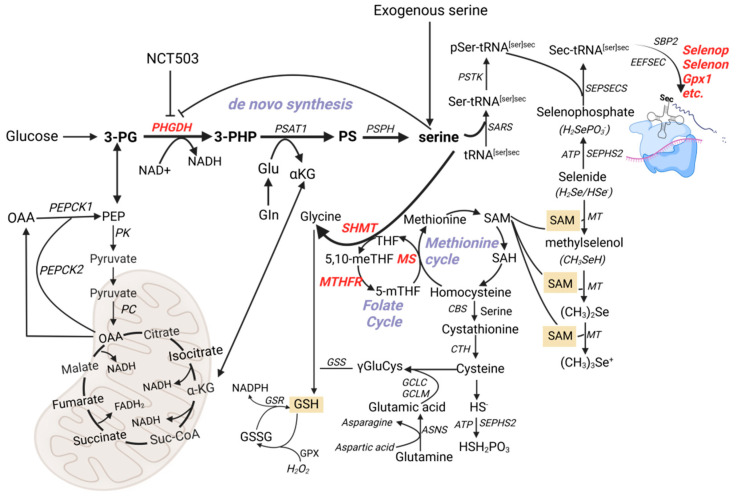
The SSP supports selenium detoxification and metabolic balance. The SSP plays a pivotal role in maintaining redox balance, supporting methylation reactions, and facilitating selenium detoxification under high selenium conditions. Increased PHGDH expression drives serine production, replenishing SAM for methylation and promoting GSH synthesis to combat oxidative stress. Excessive SSP activation burdens the methionine and folate cycles, depleting SAM and disrupting methylation homeostasis. Serine supplementation alleviates these effects by restoring one-carbon metabolism and enhancing selenium detoxification. Similarly, pharmacological inhibition of PHGDH (e.g., NCT-503) reduces SSP overactivation, highlighting its potential as a therapeutic target in Se-induced metabolic dysregulation.

Our study found that high-Se conditions significantly upregulated key enzymes involved in the methionine and folate cycles, including SHMT1 in the liver and pancreas (Figure 3e and Figure 5d), MTHFR in the liver and muscle (Figure 4f and Figure 5f), and MS in the liver, muscle, and pancreas (Figure 4g, Figure 5g and Figure 6e). These results were consistent with our previous observations that endogenous serine synthesis through SSP contributes to methyl group supply, facilitating SAM regeneration and subsequent selenium detoxification [14]. As Figure 10 shows, serine’s dual role in redox balance and one-carbon metabolism underscores its central importance in mitigating selenium toxicity [30]. By providing one-carbon units through tetrahydrofolate intermediates, serine replenishes SAM, supports methylation reactions, and alleviates Se-induced oxidative stress. Additionally, serine enhances antioxidant defenses by promoting GSH synthesis via the transsulfuration pathway [31]. Interestingly, the observed upregulation of MS and SHMT1 was significantly inhibited by exogenous serine or NCT-503 (Figure 4g, Figure 5g and Figure 6d), which reveals that serine supplementation not only mitigates SSP overactivation but also restores methionine and folate cycle functions, alleviates metabolic burdens, and reduces selenium toxicity. Notably, pharmacological inhibition of PHGDH with NCT-503 achieves similar effects, further supporting SSP as a promising therapeutic target in managing selenium-induced metabolic dysfunction.

**Figure 11:**
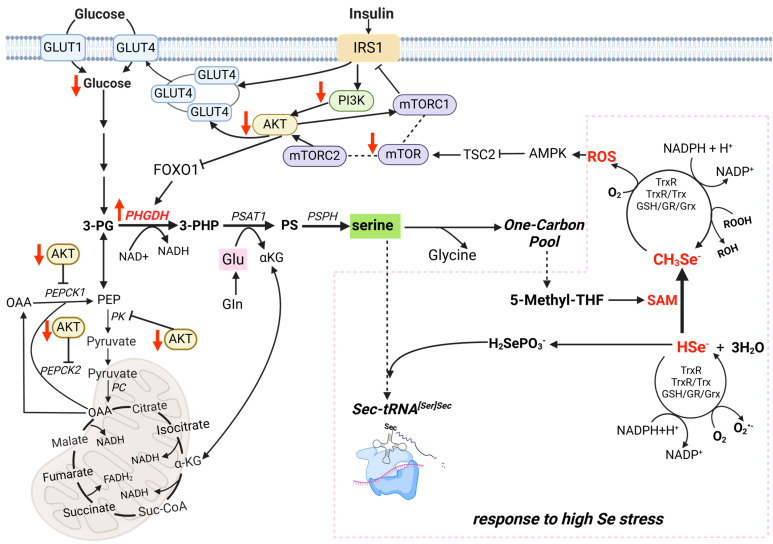
Integration of mTOR signaling, oxidative stress, and IR. The figure delineates the intricate mechanisms by which mTOR signaling integrates oxidative stress, energy homeostasis, and insulin sensitivity. Oxidative stress activates AMPK, which inhibits mTORC1 via TSC2 phosphorylation and suppresses Rheb, a key mTOR activator. Reduced mTOR signaling impairs AKT activation, disrupting GLUT4 translocation and diminishing glucose uptake. Concurrently, impaired AKT fails to suppress FOXO1 nuclear activity, leading to enhanced gluconeogenesis through the upregulation of key enzymes like PEPCK. Elevated oxidative stress and inflammation further aggravate IR by activating pathways, inducing aberrant IRS1 phosphorylation, and reducing insulin receptor sensitivity. These combined effects underscore the centrality of mTOR in maintaining redox balance, metabolic stability, and insulin signaling integrity.

As we discussed in a previous study [14], Se-induced metabolic stress activates the SSP through upregulation of the rate-limiting enzyme PHGDH, leading to increased serine synthesis. However, excessive selenium consumption may place a substantial metabolic burden on redox balance. The activation of the PI3K-AKT-mTOR pathway is important for glucose intake [32,33,34]. Interestingly, the regulation of PHGDH and mTOR under high-Se stress follows distinct and independent pathways (Figure 11). While mTOR activity is attenuated by AMPK in response to energy and oxidative stress, SSP activation via PHGDH upregulation is primarily driven by ATF4 and FOXO1 [35]. As observed in this study, high-Se levels significantly downregulated phosphorylation of AKT at Ser473 and Thr308 (Figure 7d, Figure 8d and Figure 9d,e), diminished mTOR expression (Figure 8b and Figure 9b), and reduced PI3K activity (Figure 8f), collectively indicating compromised pathway functionality. Supplementation with exogenous serine or NCT-503 enhanced the expressions of mTOR in the liver and pancreas (Figure 7b and Figure 9b and P-AKT in the pancreas (Figure 9d,e), which implied alleviating SSP overactivation and restoring PI3K-AKT-mTOR pathway activity.

The dose of serine in this study is 240 mg/kg body weight, which is also close to previous studies, such as the reported 280 mg/kg dose that ameliorated diabetic symptoms in murine models [36]. In healthy adults (average body weight 70 kg), the no-observed-adverse-effect-level (NOAEL) for L-serine is 12 g/day, corresponding to a daily intake of 171 mg/kg body weight [37]. According to the US FDA, L-serine, as a food additive, is considered safe when not exceeding 8.4% of total dietary protein (67.2 mg/kg body weight). For specific conditions (e.g., diabetes) or high-demand scenarios, L-serine supplementation can reach up to 30 g/day, equal to 429 mg/kg body weight [38]. Future studies should focus on characterizing the dose–response curve for serine supplementation, particularly at lower doses, to identify the minimal effective dose. Additionally, investigations should explore the appropriate equivalent dosing for humans and evaluate its safety and efficacy in clinical settings. Such research will be essential for refining dietary and therapeutic guidelines for serine use in mitigating excess Se-induced glucose metabolic dysregulation.

Despite the comprehensive investigation of high Se-induced IR and the mitigating effects of serine supplementation and PHGDH inhibition, several limitations remain in this study. First, our findings are based on C57BL/6J mice, which may not fully capture interspecies differences or directly translate to human physiology. Second, while the 5-month duration of selenium exposure and serine supplementation was sufficient for experimental observations, it does not reflect chronic long-term exposure scenarios in humans. Furthermore, additional studies involving a broader range of animal models and a detailed exploration of serine dosage are required to better understand its application and establish effective and safe dosing guidelines for potential human use.

## 5. Conclusions

In this study, we provide further evidence for the pivotal role of the SSP in high Se-induced glucose metabolic dysfunction. Our study observed the effects of serine supplementation to mitigate SSP overactivation, modulate methionine and folate cycles, and reactivate the PI3K-AKT-mTOR signaling pathway. By evaluating serine as both a dietary and therapeutic agent, this study could establish a foundation for translational applications in addressing metabolic disorders related to excessive selenium exposure, including IR and diabetes.

## Figures and Tables

**Table 1:** Diet composition with selenium content (unit/kg) for mice.

	Adequate-Se (0.1 mg/kg)	High-Se (0.8 mg/kg)
L-alanine	0.35	0.35
L-arginine	1.21	1.21
L-asparagine	0.6	0.6
L-aspartic acid	0.35	0.35
L-cysteine	0.35	0.35
L-glutamic acid	4	4
Glycine	2.33	2.33
L-his	0.45	0.45
L-isoleucine	0.82	0.82
L-methionine	0.82	0.82
L-leucine	1.11	1.11
L-Lysine hydrochloride	1.8	1.8
L-Phenylalanine	0.75	0.75
L-proline	0.35	0.35
L-serine	0.35	0.35
L-threonine	0.82	0.82
L-Tryptophan	0.18	0.18
L-Tyrosine	0.5	0.5
L-valine	0.82	0.82
Corn starch	15	15
Maltodextrin	15	15
Sucrose	35.17	35.17
Cellulose	3	3
Soybean oil	8	8
Choline Bitartrate	0.25	0.25
Calcium hydrogen phosphate	0.82	0.82
AIN mineral mixture	3.5	3.5
AIN Vitamin Mix	1.3	1.3
Sodium selenite	0.02190 g	0.175267 g
Total	100	100

## Data Availability

The data presented in this study are experimental data generated during the research. These experimental data are available in the Supplementary Materials.

## Supplementary Materials

The following supporting information can be downloaded at: https://www.mdpi.com/article/10.3390/nu17020311/s1, Table S1: Glucose levels, body weight, OGTT, and ITT area under the curve (AUC) results; Table S2: Changes in plasma biochemical markers (insulin, homocysteine and serine); Table S3: Selenium Levels in Mouse Plasma, Liver, Muscle, and Pancreas (µg/kg); Table S4: PHGDH Enzyme Activity in Mouse Liver, Muscle, and Pancreas (U/g); Table S5: Analysis of Western Blot Grayscale Values in Mouse Liver; Table S6: Analysis of Western Blot Grayscale Values in Mouse Muscle; Table S7: Analysis of Western Blot Grayscale Values in Mouse Pancreas.
