# Supplementary material for: The Inhibitory Effects of NCT503 and Exogenous Serine on High-Selenium Induced Insulin Resistance in Mice
Source: Nutrients. 2025 Jan 16;17(2):311. doi: 10.3390/nu17020311 (PMC11767638; doi:10.3390/nu17020311)
Supplement: Supplementary file 1 [file nutrients-17-00311-s001.zip › nutrients-3310011-supplementary.pdf]

Note: **a** indicates a significant difference from the 0.1 mg Se/kg group, **b** from the 0.8 mg Se/kg group, and **c** from the 0.8 mg Se/kg + Ser group. Significance levels are: 1 (P ⩽ 0.0332), 2 (P ⩽ 0.0021), 3 (P ⩽ 0.0002), and 4 (P ⩽ 0.0001). In the 0.8 mg Se/kg group, data marked in red indicate a significant increase compared to the 0.1 mg Se/kg group. Data marked in green in the Serine and NCT503 intervention groups show a significant decrease compared to the high Se group, while red markings in these groups indicate a significant increase compared to the high Se group.

Table S1. Glucose levels, body weight, OGTT, and ITT area under the curve (AUC) results

	0.1mg Se/kg	0.8mg Se/kg	0.8mg Se/kg+Ser	0.8mg Se/kg+NCT503
Glucose (mmol/L)	32.33±3.22	36.6±2.12(a2)	24.77±4.23 (b4)	28.74±1.81 (b4c1)
Weight (g)	608.9±42.71	618.7±35.62	532.9±88.84 (b2)	606.5±13.81 (c1)
OGTT (mmol/L)	132.4±11.99	137.6±12.13	109.8±17.34 (b3)	111.8±18.21 (b2)
ITT (mmol/L)	1473±300.9	2017±421.3 (a3)	2343±228.8	2254±263

Table S2. Changes in plasma biochemical markers (insulin, homocysteine and serine)

	0.1mg Se/kg	0.8mg Se/kg	0.8mg Se/kg+Ser	0.8mg Se/kg+NCT503
Ins (μU/mL)	9.048±0.238	15.12±0.533 (a4)	10.58±0.406(b4)	10.93±0.853 (b4)
Hcy (μmol/L)	11.8±0.601	10.1±0.422 (a3)	8.367±0.641	12.17±0.766 (b4c4)
Serine (μmol/L)	63.19±5.177	76.3±2.056 (a4)	79.65±3.509	39.78±2.927 (b4c4)

Table S3. Selenium Levels in Mouse Plasma, Liver, Muscle, and Pancreas (μg/kg)

	0.1mg Se/kg	0.8mg Se/kg	0.8mg Se/kg+Ser	0.8mg Se/kg+NCT503
Plasma	182±31.05	459.7±41.62 (a4)	459.9±31.71	468.1±37.65
Liver	1036±82.72	1857±59.56 (a4)	1845±71.35	1823±52.01
Muscle	138.60±16.15	360.60±28.67 (a4)	352.70±27.10	365.20±31.79
Pancreas	178.60±16.70	413.20±35.48 (a4)	400.30±14.47	404.20±30.07

Table S4. PHGDH Enzyme Activity in Mouse Liver, Muscle, and Pancreas (U/g)

	0.1mg Se/kg	0.8mg Se/kg	0.8mg Se/kg+Ser	0.8mg Se/kg+NCT503
Liver	8.162±0.647	12.360±0.614 (a4)	9.125±0.406 (b4)	6.578±0.134 (b4c4)
Muscle	5.148±0.240	8.156±0.458 (a4)	5.848±0.595 (b4)	3.809±0.309 (b4c4)
Pancreas	5.986±0.439	9.969±0.728 (a4)	7.734±0.524 (b4)	5.157±0.259 (b4c4)

Table S5. Analysis of Western Blot Grayscale Values in Mouse Liver

	0.1mg Se/kg	0.8mg Se/kg	0.8mg Se/kg+Ser	0.8mg Se/kg+NCT503
GPX1	1.148±0.631	3.363±0.416 (a1)	3.532±0.538	1.827±0.890 (c1)
SELENOP	3.365±0.143	4.486±0.472(a1)	4.511±0.568	3.336±0.174 (b1c1)
PHGDH	2.100±0.339	3.680±0.504 (a1)	2.363±0.643	3.927±0.541 (c1)
SHMT1	1.044±0.081	2.207±0.255 (a2)	1.552±0.453	1.956±0.325
MTHFR	1.223±0.417	2.899±0.0415 (a2)	2.338±0.372	2.840±0.735
MS	1.332±0.386	2.900±0.160 (a3)	1.833±0.293 (b2)	2.316±0.279
mTOR	1.755±0.180	1.186±0.036	1.889±0.177 (b1)	1.941±0.467 (b1)
AKT	1.127±0.113	0.899±0.098	1.306±0.133	1.302±0.346
p-AKT(ser-473)	3.553±0.489	1.625±0.204 (a3)	2.020±0.054	1.844±0.143
p-AKT(thr-308)	2.896±0.356	2.294±0.145	2.921±0.070	2.167±0.619
PI3K	2.310±0.277	1.851±0.090	2.291±0.240	1.899±0.386

Table S6. Analysis of Western Blot Grayscale Values in Mouse Muscle

	0.1mg Se/kg	0.8mg Se/kg	0.8mg Se/kg+Ser	0.8mg Se/kg+NCT503
GPX1	0.608±0.115	1.147±0.104 (a3)	1.178±0.055	0.880±0.098 (b1c1)
SELENOP	0.640±0.130	1.182±0.074 (a2)	1.376±0.038	0.844±0.196 (b1c2)
PHGDH	0.364±0.043	1.133±0.363	0.598±0.099	1.347±0.542
SHMT1	1.872±0.908	2.557±0.217	1.664±0.139	2.261±0.187
MTHFR	0.989±0.209	2.091±0.643 (a1)	1.559±0.116	1.848±0.081
MS	1.130±0.267	1.917±0.167 (a2)	0.666±0.095 (b3)	0.998±0.233 (b2)
mTOR	0.912±0.054	0.600±0.041 (a1)	0.773±0.085	0.650±0.160
AKT	1.936±0.297	1.573±0.058	1.729±0.062	1.664±0.245
p-AKT(ser-473)	1.597±0.300	0.963±0.014 (a1)	1.173±0.133	1.238±0.269
p-AKT(thr-308)	1.824±0.601	0.858±0.156	1.594±0.546	1.689±0.282
PI3K	1.589±0.383	0.832±0.085 (a1)	1.249±0.222	1.239±0.281

Table S7. Analysis of Western Blot Grayscale Values in Mouse Pancreas

	0.1mg Se/kg	0.8mg Se/kg	0.8mg Se/kg+Ser	0.8mg Se/kg+NCT503
GPX1	0.618±0.079	0.840±0.022	0.921±0.152	0.420±0.038 (b2c3)
PHGDH	0.713±0.262	1.844±0.180 (a3)	1.449±0.048	2.073±0.118 (c2)
SHMT1	0.647±0.088	1.173±0.109 (a1)	0.716±0.136 (b1)	1.268±0.224 (c2)
MTHFR	0.703±0.147	0.826±0.048	0.681±0.067	0.852±0.063
MS	0.707±0.092	1.259±0.166 (a2)	1.026±0.064	1.311±0.225
mTOR	0.980±0.061	0.627±0.069(a3)	1.012±0.063（b3）	1.043±0.039 (b3)
AKT	0.789±0.273	0.560±0.079	0.820±0.070	0.844±0.047
p-AKT(ser-473)	1.910±0.093	1.264±0.144 (a3)	1.974±0.109 (b3)	1.836±0.063 (b3)
p-AKT(thr-308)	0.630±0.050	0.414±0.070(a1)	0.590±0.103	0.700±0.059 (b2)
PI3K	0.462±0.072	0.289±0.030	0.420±0.107	0.508±0.106
